# *Zea mays i*RS1563: A Comprehensive Genome-Scale Metabolic Reconstruction of Maize Metabolism

**DOI:** 10.1371/journal.pone.0021784

**Published:** 2011-07-06

**Authors:** Rajib Saha, Patrick F. Suthers, Costas D. Maranas

**Affiliations:** Department of Chemical Engineering, The Pennsylvania State University, University Park, Pennsylvania, United States of America; Technical University of Denmark, Denmark

## Abstract

The scope and breadth of genome-scale metabolic reconstructions have continued to expand over the last decade. Herein, we introduce a genome-scale model for a plant with direct applications to food and bioenergy production (i.e., maize). Maize annotation is still underway, which introduces significant challenges in the association of metabolic functions to genes. The developed model is designed to meet rigorous standards on gene-protein-reaction (GPR) associations, elementally and charged balanced reactions and a biomass reaction abstracting the relative contribution of all biomass constituents. The metabolic network contains 1,563 genes and 1,825 metabolites involved in 1,985 reactions from primary and secondary maize metabolism. For approximately 42% of the reactions direct literature evidence for the participation of the reaction in maize was found. As many as 445 reactions and 369 metabolites are unique to the maize model compared to the AraGEM model for *A. thaliana*. 674 metabolites and 893 reactions are present in *Zea mays i*RS1563 that are not accounted for in maize C4GEM. All reactions are elementally and charged balanced and localized into six different compartments (i.e., cytoplasm, mitochondrion, plastid, peroxisome, vacuole and extracellular). GPR associations are also established based on the functional annotation information and homology prediction accounting for monofunctional, multifunctional and multimeric proteins, isozymes and protein complexes. We describe results from performing flux balance analysis under different physiological conditions, (i.e., photosynthesis, photorespiration and respiration) of a C4 plant and also explore model predictions against experimental observations for two naturally occurring mutants (i.e., *bm1* and *bm3*). The developed model corresponds to the largest and more complete to-date effort at cataloguing metabolism for a plant species.

## Introduction

*Zea mays*, commonly known as maize or corn, is a plant organism of paramount importance as a food crop, biofuel production platform and a model for studying plant genetics [1]. Maize accounts for 31% of the world production of cereals occupying almost one-fifth of the worldwide land dedicated for cereal production [2]. Maize cultivation led to 12 billion bushels of grain in the USA alone in 2008 worth $47 billion [3]. Maize is the second largest crop, after soybean, used for biotech applications [2]. In addition to its importance as a food crop, 3.4 billion gallons of ethanol was produced from maize in 2004 [3]. Maize derived ethanol accounts for 99% of all biofuels produced in the United States [3]. However, currently nearly all of this bioethanol is produced from corn seed [4]. Ongoing efforts are focused on developing and commercializing technologies that will allow for the efficient utilization of plant fiber or cellulosic materials (e.g. maize stover and cereal straws) for biofuel production. Maize is the most studied species among all grasses with respect to cell wall lignification and digestibility, which are critical for the efficient production of cellulosic biofuels [5]. A thorough evaluation of the metabolic capabilities of maize would be an important resource to address challenges associated with its dual role as a food (e.g., starch storage) and biofuel crop (e.g., cell wall deconstruction).

This decade we witnessed significant advancements towards mapping plant genes to metabolic functions culminating with the complete genome sequencing and partial annotation of a number of plant species, namely, *Arabidopsis thaliana* [6], *Oryza Sativa* [7], [8], *Sorghum bicolor* [9], *Zea mays* [10] and *Theobroma cacao* [11]. Nevertheless, attempts to engineer plant metabolism for desired overproductions have been met with only limited success [12]. Genetic modifications seldom bring about the expected/desired effect in plant metabolism primarily due to the built-in metabolic redundancy circumventing the imposed genetic changes [13], [14]. This necessitates the development of genome-wide comprehensive metabolic reconstructions capable of taking account of the complete inventory of metabolic transformations of a given plant organism.

Genome-scale metabolic reconstructions are available for an increasing number of organisms [15], [16]. At least 40 bacterial, 2 archaeal and 15 eukaryotic reconstructions are available to-date [12], [15], [17], [18] while many others are under development. Recently Poolman et al (2009) and Dal'Molin et al (2010) independently constructed the first two genome-scale metabolic reconstructions for a plant organism (i.e., *Arabidopsis thaliana*). The model by Dal'Molin *et al* identifies the set of essential reactions, accounts for the classical photorespiratory cycle and highlights the significant differences between photosynthetic and non-photosynthetic metabolism. The model by Poolman *et al* includes ATP demand constraints for biomass production and maintenance and suggests strategies for the construction of metabolic modules as a consequence of variation in ATP requirement. Both models make a significant step forward towards assessing the metabolic capabilities of plants establishing production routes for key biomass precursors and major pathways of Arabidopsis primary metabolism. In addition, two recent efforts involved the reconstruction of plant models with an emphasis on specific physiological conditions or tissue types [19], [20]. Model C4GEM [20] focused on C4 plants such as maize, sugarcane and sorghum and investigated flux distributions in mesophyll and bundle sheath cells during C4 photosynthesis. Grafahrend-Belau *et al* developed a metabolic network of only primary metabolism in barley seeds and studied grain yield and metabolic fluxes under a variety of oxygen availability scenarios and genetic manipulations [19]. Pilalis *et al.* reconstructed a multi-compartmental model of the central metabolism of *Brassica napus* (Rapeseed) and simulated seed growth during the stage of oil accumulation and subsequently studied network properties of seed metabolism via Flux Balance Analysis, Principal Component Analysis and reaction deletion studies [21].

In this paper, we describe the construction of a genome-scale in silico model of maize metabolism (i.e., *Zea mays i*RS1563). This is, to the best of our knowledge, the first attempt of globally characterizing the metabolic capabilities (both primary and secondary metabolism) using a compartmentalized photosynthetic model of an important crop and energy plant species. The development of a genome-scale model for maize is a significant challenge due to its genome size which is 14 times larger [10] than that of *Arabidopsis thaliana* (157 million base pairs) [22]. The constructed model contains 1,563 genes and 1,825 metabolites participating in 1,985 reactions from both primary and secondary metabolism of maize. For 42% of the reaction entries direct literature evidence in addition to homology criteria for their inclusion to the model was identified. We found that as many as 676 reactions and 441 metabolites are unique to *Zea mays i*RS1563 in comparison to the AraGEM model by Dal'Molin *et al*. We chose the AraGEM model as a basis of comparisons as at the onset of this study it was the most comprehensive genome-scale compartmentalized model of a plant species capable of recapitulating basic plant physiological states. In order to deduce the genuine differences between maize and Arabidopsis irrespective of annotation chronology we also reconstructed an up-to-date model of Arabidopsis, *A. thaliana i*RS1597. *A. thaliana i*RS1597 contains 1597 genes, 1798 reactions and 1820 metabolites. In comparison to *A. thaliana i*RS1597, *Zea mays i*RS1563 has 445 new reactions and 369 new metabolites. Notably, 893 reactions and 674 metabolites are included in *Zea mays i*RS1563 that are absent from the maize C4GEM model. All reactions present in Zea mays iRS1563 are elementally and charged balanced and localized into six compartments including cytoplasm, mitochondrion, plastid, peroxisome, vacuole and extracellular space. Provisions for accounting that photosynthesis in maize (i.e., a C4 plant) occurs in two separate cell types (i.e., mesophyll cell and bundle sheath cell) are included in the model. GPR associations are delineated from the available functional annotation information and homology prediction accounting for monofunctional, multifunctional and multimeric proteins, isozymes and protein complexes. A biomass equation is established that quantifies the relative abundance of different constituents of dry plant cell biomass. Biomass production under three different physiological states (i.e., photosynthesis, photorespiration and respiration) is demonstrated and the model is tested against experimental data for two naturally occurring maize mutants (i.e., bm1 and bm3).

## Results

The metabolic model reconstruction process follows three major steps: (1) Reconstruction of draft model via automated homology searches for the identification of native biotransformations; (2) Generation of a computations-ready model after defining biomass equation and system boundary and establishing GPR; (3) Model refinement via GapFind and GapFill [23] to unblock biomass precursors as well as reconnect unreachable metabolites. Upon construction of the model, key features such as physiological constraints, network connectivity, light reactions, carbon fixation and secondary metabolism and uniqueness compared to AraGEM and maize C4GEM are described. In addition, model predictions are contrasted against experimental observations.

### Construction of Auto and Draft models

The B73 maize genome [10] has 32,540 genes and 53,764 transcripts in the Filtered Gene Set (FGS). Out of 32,540 genes, 30,599 (93%) are evidence-based [24], while the remaining 2,141 (7%) are predicted by the Fgenesh program [25]. 13,726 genes (42% of total) do not have any functional annotation information or are identified as proteins with no or hypothetical/putative functions. Of the remainder, 1,361 (7%) genes encode proteins that do not participate in specific metabolic transformations but rather are involved in transcription, signal transduction, DNA repair, DNA binding, DNA/RNA polymerization, protein folding and adhesion. Because the B73 maize genome is not completely annotated we first established Gene-Protein-Reaction (GPR) mappings for the AraGEM genome-scale model of *A. thaliana* [12] to be used as a proxy. Using these GPRs as a point of comparison we next identified Arabidopsis gene orthologs in maize and transferred the corresponding GPRs via the AUTOGRAPH method [26]. This step was followed by annotation of the remainder maize genes by bidirectional protein BLAST (i.e., BLASTp) searches against the NCBI non-redundant (nr) database. Out of a total of 1,567 metabolic or transport reactions of AraGEM, GPRs were established for 1,254 reactions via 1,467 genes and 653 enzymes by making use of information from several online databases such as AraCyc, KEGG, Uniprot and Brenda (see File S1). Bidirectional BLASTp searches for each one of the 1,467 genes included in AraGEM model were carried out against the B73 maize genome using a stringent cutoff value of 10^−30^. This fully automated process generated an initial model, termed as *‘Automodel’*, containing 946 genes and 1,365 unique metabolites participating in 1,186 reactions (see Table 1 and File S2) exclusively derived from AraGEM. Out of 1,186 reactions, 32 are inter-organelle transport reactions for which homologs were found in maize.

**Table 1:** Model size after each reconstruction step.

	Auto model	Draft model	Functional model	Final model
**Included genes**	946	1,485	1,552	1,563
**Proteins**	472	714	774	876
Single functional proteins	178	322	381	463
Multifunctional proteins	92	150	153	170
Protein complexes	0	4	4	4
Isozymes	21	36	36	36
Multimeric proteins	87	140	148	148
Others^a^	94	62	62	55
**Reactions**	1,186	1,667	1,821	1,985
Metabolic reactions	1,154	1,635	1,739	1,900
Transport reactions	32	32	67	70
*GPR associations*				
Gene associated (metabolic/transport)	1,100	1,581	1,635	1,668
Nonenzyme associated (metabolic/transport)	86	86	86	86
Spontaneous^b^	0	0	7	41
Nongene associated (metabolic/transport)	0	0	78	175
Exchange reactions	0	0	15	15
**Metabolites** c	1,365	1,703	1,769	1,825
Cytoplasmic	1,309	1,643	1,689	1,744
Plastidic	91	102	114	115
Peroxisomic	67	69	92	93
Mitochondrial	60	82	86	86
Vaccuolic	5	5	5	5
Extracellular	0	0	15	15

a Others include proteins involve in complex relationships, e.g. multiple proteins act as protein complex which is one of the isozymes for any specific reaction.

b Spontaneous reactions are those without any enzyme as well as gene association.

c Unique metabolites irrespective of their compartmental location.

**Figure 1:**
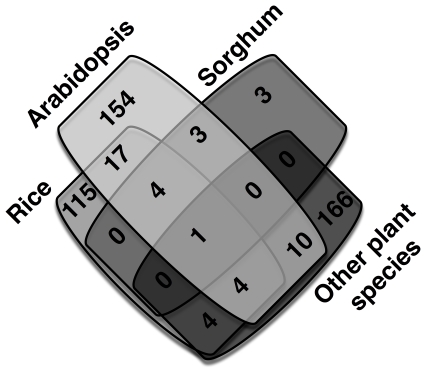
Species origin of newly added reactions in the Draft model.

Genes not included in the automodel were scrutinized further by comparing them against the NCBI non-redundant protein database using the same BLASTp cut-off. This increased the model size to 1,485 genes and 1,703 unique metabolites involved in 1,667 reactions by pulling functionalities absent in AraGEM. This is referred to as the *‘Draft model’* (see Table 1 and Files S2 and S3). As described in Table 2, orthologous genes were found in *Oryza Sativa* (Rice), *Arabidopsis thaliana* (Arabidopsis), *Sorghum bicolor* (Sorghum) and less frequently in other plant species such as wheat, tobacco, spinach, soya bean, etc. (See File S3). Notably, 802 orthologous genes from *A. thaliana* were added in the model *Zea mays i*RS1563 that were absent from AraGEM primarily due to recent annotation updates. Reactions associated with these genes were subsequently extracted from on-line databases such as KEGG and BRENDA. Table 2 shows the total number of reactions as well as the number of new reactions included in the draft model. Seven reactions having KEGG reaction IDs R00379, R00381, R06023, R06049, R06082, R06138 and R06209 were excluded since they involve generic groups and were not elementally fully defined. Figure 1 shows the distribution of the newly added reactions in the draft model based on their orthologous gene of origin.

**Table 2:** Maize gene annotation via bidirectional BLASTp homology searches against NCBI non-redundant protein database.

Species	Number of orthologs	Number of associated reactions	Number of newly added reactions in draft model
*Oryza Sativa* (Rice)	4,109	312	145
Other plant species	833	214	185
*Arabidopsis Thaliana (Arabidopsis)*	802	258	193
*Sorghum Bicolor* (sorghum)	47	20	11

### Generation of computations-ready model

A computations-ready model requires a fully characterized biomass equation, assignment of metabolites to reactions, establishment of GPR associations, localization of reactions in compartment(s), and inclusion of intra- and extracellular transport reactions [27].

#### (i) Establishing a fully characterized biomass equation

A biomass equation that drains all necessary precursors present in maize was derived (see File S4 and Table 3). We used the biomass composition of young and vegetative maize plants as measured by Penningd *et al.* and expressed on a dry weight basis [28]. The amino acid and lignin composition were derived based on the data from [29], [30]. The composition of hemicellulose was approximated using data for Orchard Grass [31], another monocot grass species, as no corresponding information was found for maize. Based on these compositions we also defined aggregate reactions such as ‘Amino acid synthesis’, ‘Protein synthesis’, ‘Carbohydrate synthesis’, ‘Hemicellulose synthesis’, ‘Lignin synthesis’, ‘Lipid synthesis’, ‘Material synthesis’, ‘Nitrogenous compound synthesis’, ‘Nucleic acid synthesis’ and ‘Organic acid synthesis’ to produce necessary biomass precursors (i.e., amino acids, protein, carbohydrates, hemicellulose, lignin, lipids, materials, nitrogenous compounds, nucleic acids and organic acids respectively). The biomass equation also contains a non-growth associated ATP maintenance as in the latest Arabidopsis model AraGEM [12].

**Table 3:** Biomass component list in *i*RS1563.

Major components	Protein	Carbohydrates	Lipids	Ions
**Nitrogenous compounds**	L-alanine	ribose	glyceroltripalmitate	potassium
**Carbohydrates**	L-arginine	glucose	gleceroltristearate	chloride
**Lipids**	L-aspartic acid	fructose	glyceroltrioleate	
**Lignin**	L-cystine	mannose	glyceroltrilinolate	**RNA**
**Organic acids**	L-glutamic acid	galactose	glyceroltrilinoleate	ATP
**Ions**	L-glycine	sucrose		GTP
	L-histidine	cellulose	**Lignin**	CTP
	L-isoleucine	hemicellulose	4-coumaryl alcohol	UTP
**Nitrogenous compounds**	L-leucine	pectin	coniferyl alcohol	
amino acids	L-lysine		sinapyl alcohol	**DNA**
protein	L-methionine			dATP
nucleic acids	L-phenylalanine	**Hemicellulose**	**Organic acids**	dGTP
	L-proline	arabinose	oxalic acid	dCTP
	L-serine	xylose	glyoxalic acid	dUTP
	L-threonine	mannose	Oxalo-acetic acid	
	L-tryptophan	galactose	Malic acid	
	L-tyrosine	glucose	Citric acid	
	L-valine	uronic acids	aconitic acid	

#### (ii) Assignments of genes, reactions, metabolites and compartments

All metabolic and inter-organelle transport reactions in the draft model have full gene associations. During this step all reactions were elementally balanced and metabolites were assigned appropriate protonation states corresponding to a physiological pH of 7.2. We included an additional 86 reactions to the model without enzyme association information based on direct literature evidence [12]. For example, reactions with KEGG IDs R08053, R08054 and R08055 involved in chlorophyll metabolism are included in the model. Reaction localization information for maize can in some cases be found in database PPDB (a plant proteome database of maize and Arabidopsis) [32]. Because only limited reaction localization information exists for maize, we adopted the compartment or organelle reaction location of the corresponding orthologous gene/enzyme in Arabidopsis using the Arabidopsis Subcellular Database, SUBA [33] and also PPDB [32]. As in AraGEM, reactions for which no such information is available we assumed that they are present only in the cytoplasm.

#### (iii) Identification of system boundary

The entire reaction network (i.e., system boundary) was distributed across five different intracellular organelles enveloped by the cytoplasmic membrane. Exchange reactions were added in the model to ensure that gaseous metabolites (i.e., carbon dioxide and oxygen), inorganic nutrient metabolites (i.e., nitrate, ammonia, hydrogen sulfide, sulfate, phosphate, potassium and chloride), sugar metabolites (i.e., glucose, fructose, maltose and sucrose), water and photons could enter and leave the system whenever necessary depending on the physiological state. As shown in Table 4, constraints on these exchange reactions as well as reactions involved with enzyme RuBisCO (Ribulose-1, 5-bisphosphate carboxylase oxygenase) were established to define three different physiological states (i.e., photosynthesis, photorespiration and respiration) by allowing the selective uptake/release of certain metabolites. Even though photorespiration is limited in C4 plants (i.e., maize, sorghum, etc.), literature evidence [34], [35], [36] alludes that it is still present. Therefore, we made sure that the model is capable of simulating this condition.

**Table 4:** Definition of three different physiological states.

Constraints	Photosynthesis(PS)	Photorespiration(PR)	Respiration(R)
CO_2_ transport	Uptake	Uptake	Release
Sucrose transport	Disabled	Disabled	Uptake
Photon transport	Uptake	Uptake	Disabled
H_2_O transport	Uptake	Uptake	Uptake
Inorganic nutrient transport	Uptake	Uptake	Uptake
O_2_ transport	Release	Unconstrained	Uptake
RUBISCO: EC 4.1.1.39	Carboxylation	Carboxylation∶Oxygenation = 3∶1	Both disabled

The stoichiometric matrix of the draft model (see Table 1) contains 1,901 rows (i.e., total metabolites after taking account of their compartmental appearance) and 1,682 columns (i.e., metabolic reactions, inter-organelle transport reactions and exchange reactions). 970 reactions have one-to-one GPR associations whereas 712 map to more than one gene. 532 reactions map to both isozymes and protein complexes while 4 of them map to only protein complexes, 36 to only isozymes, and 140 to only multimeric proteins.

### Network connectivity analysis and restoration

The draft metabolic model inherently contained gaps, unreachable metabolites, omitted transport mechanisms and missing biomass components. We used the procedures termed GapFind and GapFill [37] to correct for these pathologies. We first concentrated on resolving problems with the participation of components in the biomass equation followed by network connectivity.

We found that 723 out of the 1,683 reactions in the draft model could not carry any flux (i.e., blocked reactions) under any of the relevant three physiological states (e.g. photosynthesis (PS), photorespiration (PR) and respiration (R)). As a result, these blocked reactions prevented the formation of some of biomass precursors. GapFind [37] revealed that only 21 out of 64 biomass components could be synthesized using the draft model. GapFill [37] was applied for bridging the gaps through the addition of metabolic and inter-organelle transport reactions and the relaxing of irreversible of existing reactions in the model. GapFill suggested the addition of 94 metabolic and 35 inter-organelle transport reactions in the model to unblock the production of all 64 biomass components. These putative additions to the model were tested by performing an additional round of BLASTp searches for the corresponding genes against the maize genome. We found that 54 (out of 93) metabolic reactions could be assigned to maize gene(s) if the expectation value cut-off for BLASTp was lowered to 10^−5^. In light of the critical need of restoring biomass formation the less stringent cut-off for inclusion was accepted for these genes. Addition of these reactions ensured the production of biomass under all relevant physiological states validating the use of the term *‘Functional’* for the updated model (see Table 1).

**Figure 2:**
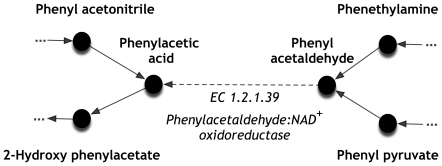
Example of connectivity restoration for phenylacetaldehyde.

Upon ensuring biomass formation GapFind was also applied to assess network connectivity and 715 blocked metabolites were found in the functional model. By applying GapFill connectivity of 322 (45%) blocked metabolites was restored through the addition of 159 metabolic and 3 inter-organelle transport reactions. Table 5 shows the distribution of blocked metabolites into four intracellular organelles before and after applying GapFill. BLASTp searches allowed us to assign 31 (20% of GapFill suggestions) metabolic reactions with specific maize genes (File S2). Biological evidence of the occurrence of such additional reactions in maize or other plant species was sought whenever possible. For example, as shown in Figure 2 phenylacetaldehyde appears to be a “no-consumption” [37] metabolite in the functional model as no reaction can consume it. Using GapFill we found a homolog in maize (i.e., BLASTp score of 10^−24^) and also literature evidence [38] that *Arabidopsis thaliana* has a aldehyde dehydrogenase activity that catalyzes the conversion of phenylacetaldehyde to phenylacetic acid. Hence, by adding this chemical transformation to *Zea mays i*RS1563 a consumption pathway for phenylacetaldehyde is established. After adding these reactions to the functional model and following charge and elemental balancing and GPR association checking the *‘Final’ Zea mays i*RS1563 model (see Table 1) is derived.

**Table 5:** Restoration of network connectivity using GapFill [36].

Number of metabolites	Number of blocked metabolites: before applyingGapFill	Number of blocked metabolites: after applyingGapFill
Cytosolic (1744)	680	382
Plastidic (115)	28	11
Peroxisomic (93)	5	0
Mitochondrial (86)	2	0

### *Zea mays i*RS1563 model

The *Zea mays i*RS1563 metabolic reconstruction contains 1,825 unique metabolites and 1,985 reactions associated with 1,563 genes and 876 proteins. Of these reactions 1,898 are metabolic reactions, 70 are inter-organelle transport reactions and 15 are exchange reactions between intra- and extracellular environments. GPR associations are established for all entries (see Table 1). Notably, we identified that the fraction of multifunctional proteins (19% of the total number of proteins) in *Zea mays i*RS1563 is similar to the ratio found in *E. coli* [39]. *Zea mays i*RS1563 accounts for the metabolic functions for all three physiological states. Photosynthetic as well as photorespiration metabolism was modelled by including light mediated ATP and NADPH production via separate charged balanced reactions in the electron transfer system of the thylakoid membrane [40]. Furthermore, the ratio of fluxes for the carboxylation and oxidation reactions associated with enzyme RuBisCO was kept at 1∶0 thus ensuring complete carbon fixation during photosynthesis. This ratio was shifted to 3∶1 during photorespiration to model simultaneous carbon fixation and oxidation [41]. Because sucrose is the main growth substrate during respiration for higher plants [42], the aforementioned reactions were inactivated and the exchange reaction for sucrose uptake was activated. Under all these three conditions, inorganic nutrients required for plant growth, e.g. sulfate, nitrate, ammonia, hydrogen sulfide, phosphate, potassium and chloride, were allowed to be freely taken up from the environment via extracellular exchange reactions.

**Figure 3:**
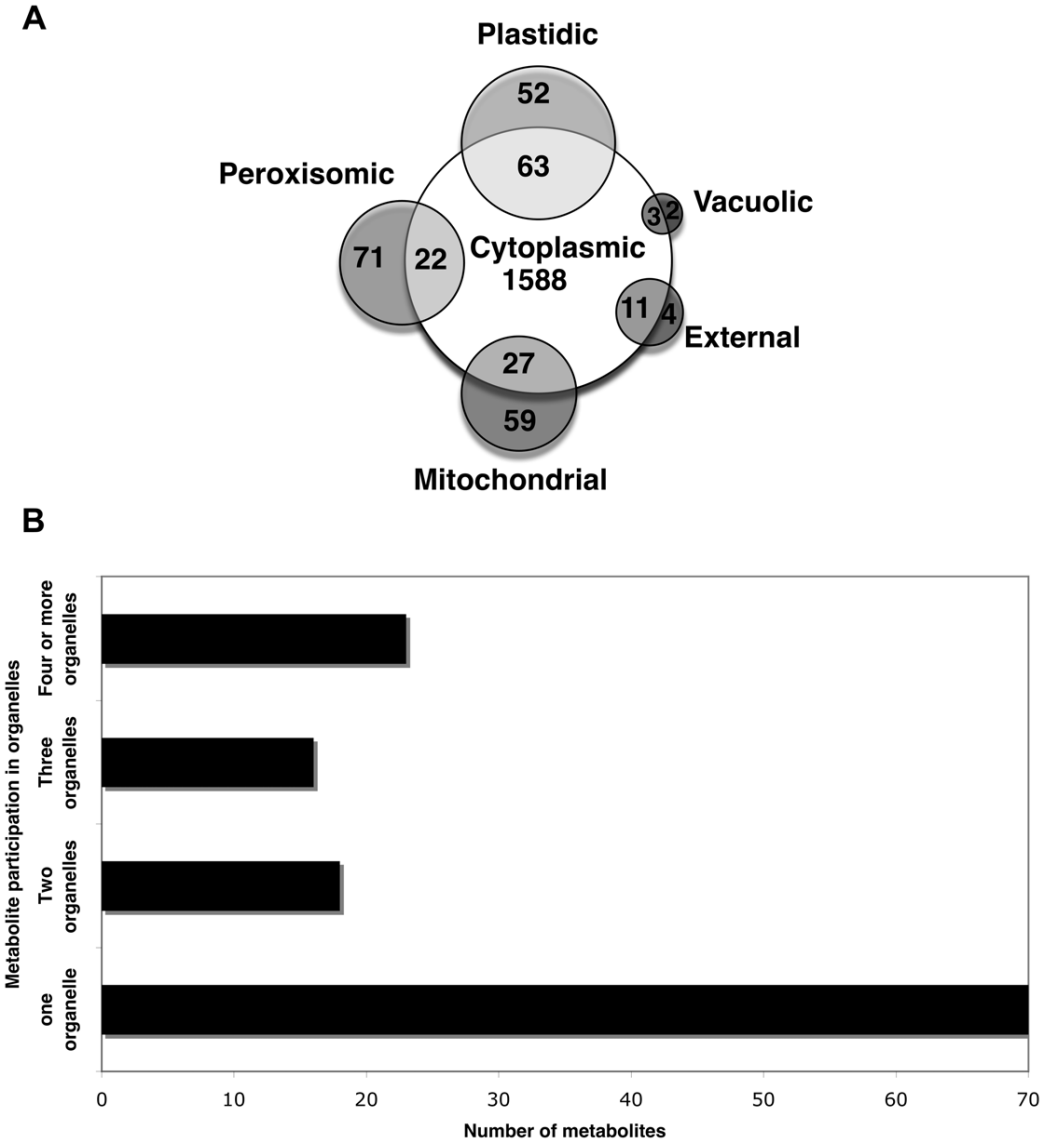
Distribution of metabolites based on their number of appearance in different organelles. (a) cytoplasmic *Zea mays i*RS1563 metabolites in cytoplasm and other organelles, and, (b) non-cytoplasmic *Zea mays i*RS1563 metabolite-organelle participation.

The participation of *Zea mays i*RS1563 metabolites across different compartments is shown in Figure 3. The five intracellular organelles differ notably in terms of mutual connectivity, metabolite uniqueness and number of metabolites. As shown in Figure 3a, approximately 90% of these metabolites are unique to cytoplasm. In addition, cytoplasm contains all metabolites shared between any two organelles because any metabolite needs to be transported through cytoplasm in order to be exchanged between organelles. Among the remaining metabolites, cytoplasm shares the highest number with the plastid (i.e., 63) where photosynthesis and photorespiration occur. It also shares a significant number of metabolites with mitochondrion (i.e., 27) and peroxisome (i.e., 22) that are involved in energy production and fatty acid biosynthesis, respectively. Figure 3b shows the distribution of other non-cytoplasmic *Zea mays i*RS1563 metabolites in terms of how many organelles they participate.

### Light reactions, carbon fixation and secondary metabolism

**Figure 4:**
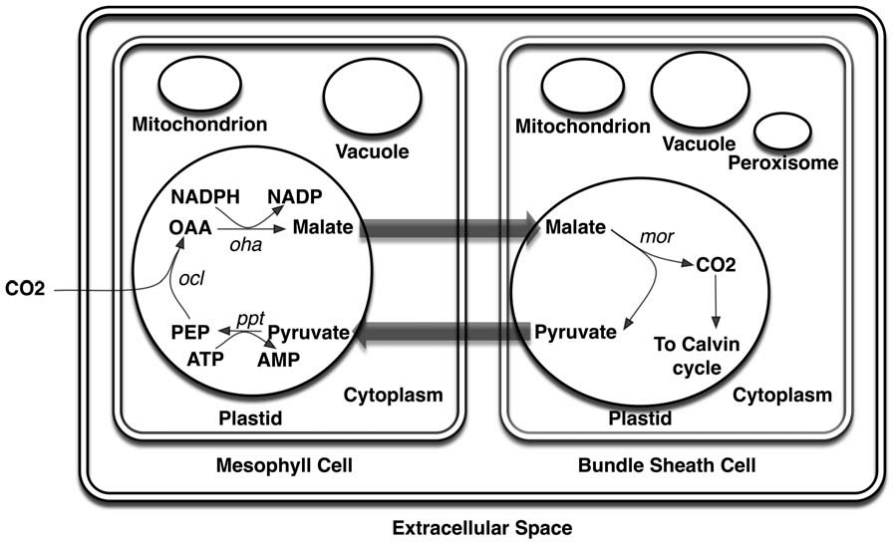
Compartment and localization information for *Zea mays iRS* 1563. Mitochondrion and vacuole compartments are present in both cell types whereas peroxisome is only present in bundle-sheath cell [40]. Plastidic reactions are distributed between mesophyll and bundle-sheath cells.

In plants photosynthesis reactions include light dependent and light independent or carbon fixation reactions [43]. *Zea mays i*RS1563 includes charged balanced light reactions culled from a number of literature sources [40], [44], [45], [46]. The overall photosynthesis reaction cascade produces two NADPH, three ATP and one O_2_ whenever nine photons are absorbed and fourteen H^+^ are transferred via the electron-transport system. This defines the following overall balance equations:
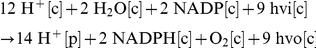


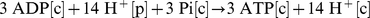
Here, [c] and [p] represent cytoplasm and plastid and hvi and hvo signify input and output photons respectively. Carbon fixation in maize (C_4_ plant) is more complex compared to Arabidopsis or other C_3_ plants [43]. *Zea mays i*RS1563 captures these differences by accounting for (i) direct carboxylation of phosphoenol pyruvate and CO_2_ fixation to form C_4_ acids such as oxaloacetic acid [ATP: oxaloacetate carboxy-lyase *(ocl)*] and malic acid [Oxaloacetate: NADPH hydrogenase *(oha)*] in mesophyll cells, (ii) transport of malic acid from mesophyll cell to bundle-sheath cells, (iii) decarboxylation of malic acid [Malate:NADP+ oxidoreductase *(mor)*] in bundle-sheath cells to produce pyruvic acid and CO_2_, which enters the Calvin cycle, (iv) transport of pyruvic acid from bundle-sheath cells to mesophyll cells, and (v) production of phosphoenol pyruvic (i.e., C_3_) acid [ATP:pyruvate,phosphate phosphotransferase *(ppt)*] from pyruvic acid [43]. Figure 4, pictorially shows the localization of reactions and organelles between mesophyll and bundle sheath cells. In addition, to differences in carbon fixation reactions, the peroxisome activity is primarily present in bundle-sheath cells and largely absent from mesophyll cells [47]. Based on this localization information a standalone metabolic model can be developed for the photosynthetic tissue of maize. Because RuBisCO that operates in the Calvin cycle cannot come in direct contact with atmospheric oxygen during day time (see Figure 4), photorespiration is restricted providing an advantage for survival in hot and arid environments for maize and other C_4_ plants. This comes at the expense of higher (ATP) requirements as C_4_ carbon fixation involves additional steps [43].

In addition to photosynthesis, secondary metabolism plays a key role in the physiology of maize. For example, phenylpropanoid metabolism produces monolignols (i.e., *p*-coumaroyl alcohol, coniferyl alcohol and sinapyl alcohol) that are used in the generation of three major lignin subunits H-lignin, G-lignin and S-lignin, respectively [48]. Many of these enzymes such as hydroxycinnamoyl transferase (HCT), ferulate 5-hydroxylase (F5H) and caffeic acid 3-*O*-methyltranferase (COMT) along with their associated reactions are unique to C_4_ plants and are not present in the lignin biosynthesis pathways of *A. thaliana* [48]. HCT is involved in the early stages of lignin biosynthesis by controlling the flux from *p*-coumaroyl-CoA towards caffeoyl-CoA while F5H and COMT regulate fluxes from coniferaldehyde and coniferyl alcohol to sinapaldehyde and sinapyl alcohol, respectively [48]. *Zea mays i*RS1563 contains all these enzymes and associated reactions thus providing a comprehensive lignin biosynthesis pathway for a C_4_ plant.

In addition to phenylpropanoid metabolism, *Zea mays i*RS1563 provides a detailed description of flavonoid biosynthesis pathways. Flavonoids are pigments occurring in plant as secondary metabolites and mostly function in the recruitment of pollinators and/or seed dispersers [49]. For example, maize is known to produce 3-deoxyanthocyanins, which are a specialized class of flavonoids [50], [51]. *Zea mays i*RS1563 contains the dihydroflavonol 4-reductase (DFR) enzyme that catalyzes the reaction for flavan-4-ols biosynthesis that channels flux towards 3-deoxyanthocyanins production [51]. The model also accounts for isoflavone 7-O-glucosyltransferase (IF7GT) and associated reactions that are involved in the production of necessary intermediates for pterocarpin phytoalexin conjugates such as medicarpin 3-O-glucoside-6′-O-malonate (MeGM) and maackain 3-O-glucoside-6′-O-malonate (MaGM) involved in plant defense against fungal elicitation [52].

### Comparing *Zea mays i*RS1563 with *Arabidopsis thaliana* and maize C4GEM models

**Figure 5:**
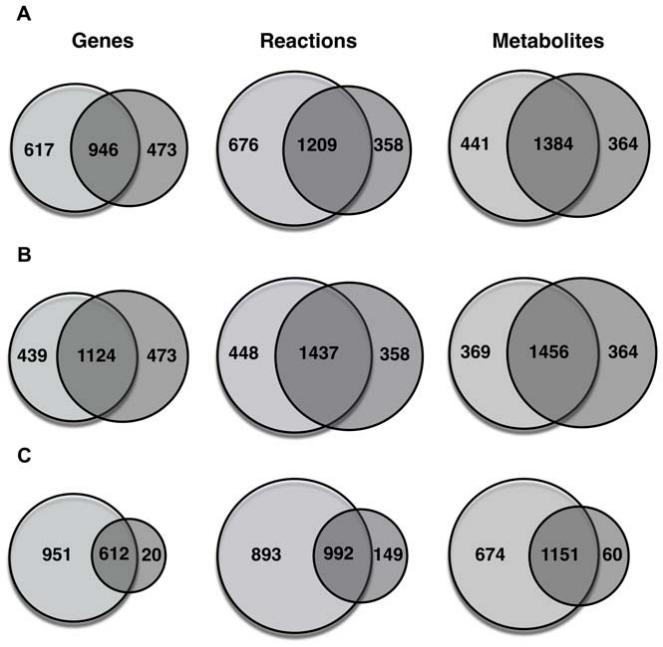
Venn diagram for genes, reactions and metabolites. (a) between *Zea mays i*RS1563 and AraGEM, (b) between *Zea mays i*RS1563 and *Arabidopsis thaliana i*RS1597, and (c) between *Zea mays i*RS1563 and maize C4GEM.

Figure 5a compares the total number of genes, reactions and metabolites between *Zea mays i*RS1563 and the *A. thaliana* AraGEM genome-scale-models [12]. Approximately, only 61% of genes in *Zea mays i*RS1563 are present in AraGEM. This yields a surprisingly low degree of matching between these two models of 64% and 76%, respectively in terms of reactions and metabolites. In the interest of elucidating the true differences between maize and Arabidopsis irrespective of annotation chronology we constructed a more up-to-date genome-scale model for Arabidopsis by appending onto AraGEM newly annotated genes as well as full GPR annotations. We refer to this updated model containing 1,597 genes, 1,798 reactions and 1,820 metabolites as *A. thaliana i*RS1597 (see File S1). The newly added 228 reactions (absent from AraGEM) are involved in various pathways in primary (i.e., glycolysis, TCA, fatty acid and amino acid biosynthesis, starch and sucrose metabolism) and secondary (i.e., biosynthesis of steroid, ubiquionone, streptomycin, thiamin, riboflavin, terpenoid, brassinosteroid, phenylpropanoid, etc.) metabolism of Arabidopsis.

A direct comparison of *Zea mays i*RS1563 with *A. thaliana i*RS1597 reveals, as expected, an increased degree of matching of 72%, 76% and 80% in terms of genes, reactions and metabolites, respectively (see Figure 5b). We find that 445 reactions are unique to maize with no counterpart in *A. thaliana*. Secondary plant metabolism including flavonoid, mono- and diterpenoid, brassinosteroid, phenylpropanoid, anthocyanin, zeatin biosynthesis, riboflavin and caffeine metabolism account for 185 of the maize-specific reactions. In addition, a variety of primary metabolism reactions dispersed throughout central metabolism, photosynthesis, amino acid and fatty acid biosynthesis account for the remaining 260 reactions. This comparison implies that about one third of the differences between *Zea mays i*RS1563 and AraGEM are caused by the incompleteness of AraGEM model especially in terms of secondary metabolism while the remaining two third reflect genuine differences between C_3_ (i.e., Arabidopsis) and C_4_ (i.e., maize) plant metabolism.

Figure 5c shows a similar comparison between *Zea mays i*RS1563 and maize C4GEM genome-scale-models. Degrees of matching between these two models are 39%, 53% and 63% in terms of genes, reactions and metabolites, respectively. This surprisingly low degree of matching is caused primarily due to the fact that maize C4GEM includes only metabolites and reactions in leaves during photosynthesis. Therefore, there are 893 reactions in *Zea mays i*RS1563 absent from maize C4GEM. 343 of these reactions describe secondary plant metabolism such as brassinosteroid, phenylpropanoid, carotenoid, flavonoid, mono- and diterpenoid, and glucosinolate metabolism. The remaining 550 reactions are found in a wide range of primary metabolism pathways such as central metabolism, photosynthesis, benjoate degradtion, starch and sucrose metabolism, lipid metabolism, nitrogen metabolism amino acid and fatty acid biosynthesis. Conversely, 116 (out of 149) new reactions in maize C4GEM have untraceable EC numbers and gene loci.

### *Zea mays i*RS 1563 model testing

*Zea mays i*RS1563 allows for the production of biomass under all three different physiological states (see Files S5 and S6 for detailed information of the model). Due to limited photorespiration C4 plants usually have higher photosynthetic efficiency [43]. Under higher light intensity and photosynthetic condition, *Zea mays i*RS1563 produces 0.0008 mole biomass/mole CO2 whereas *A. thaliana i*RS1597 yields 0.0006 mole biomass/mole CO2. Thus, the model predictions match with findings reported in literature [43]. We also investigated the model's ability to predict the effect of suppressing genes in the lignin biosynthesis pathway observed in naturally occurring *brown midrib* (*bm*) maize mutants (i.e., *bm1*, *bm2*, *bm3* and *bm4*) [48], [53], [54], [55]. These maize mutants are Mendelian recessives that are characterized by brown vascular tissue in leaves and stems due to a changed lignin content and/or composition [56]. The specific genetic background for two of these mutants (*bm1* and *bm3*) was elucidated based on the analysis of cell wall composition [55]. Mutants *bm1* and *bm3* were found to have disrupted enzymatic activity for cinnamyl alcohol dehydrogenase (CAD) and caffeic acid 3-*O*-methyltranferase (COMT). Both of these enzymes are involved in the last stages of the monolignol pathway [55] that controls lignin synthesis and composition (i.e., the ratio of three major subunits, H-lignin, G-lignin and S-lignin) [57].

We simulated mutants *bm1* and *bm3* using *Zea mays i*RS1563 under photosynthetic conditions by restricting the flux of the reactions catalyzed by enzymes CAD and COMT to 10% of the wild-type values. It is expected that the disruption of the activity for these genes will directly affect lignin content and composition (see File S7 to find literature data used for *bm1* and *bm3* mutants). We were interested to see whether the *Zea mays i*RS1563 metabolic model will be able to correctly propagate this disruption across the metabolic pathways and correctly predict the effect on other key metabolites. Table 6 contrasts experimental results by (Marita et al (2003), Vanholme et al (2008) and Sattler et al (2010)) with *in silico* predictions for the maximum theoretical yield of lignins, sugars and crude protein in terms of whether they increased, decreased, or remained the same in the mutant strains. Out of 21 compared components *Zea mays i*RS1563 correctly predicted the direction (or absence) of change for 17 cases.

**Table 6:** Change in content of cell wall components in *bm1* and *bm3* Maize mutants.

	Model findings vs Experimental observations	
	*bm1* mutant	*bm3* mutant
H-lignin	↓/ =	↓/↓
G-lignin	↓/↓	↓/↓
S-lignin	↓/↓	↓/↓
Total lignin	↓/↓	↓/↓
S-lignin/G-lignin ratio	= / =	= / =
Glucose	↓/↓	↓/↑
Mannose	↓/↓	↓/↓
Arabinose	↓/↓	↓/↓
Galactose	↓/↓	↓/↓
Xylose	↓/↑	↓/↑
Crude protein	-	↓/↓

Cell wall components include lignin subunits, total lignin, S-lignin/G-lignin ratio, sugars, starch and protein. List of used symbols include ‘↓’: decrease in quantity; ‘↑’: increase in quantity; ‘ = ’: no change in quantity, with respect to wild Maize plant; ‘/’: comparison of model findings with actual observations, and ‘-’: no experimental observation found.

**Figure 6:**
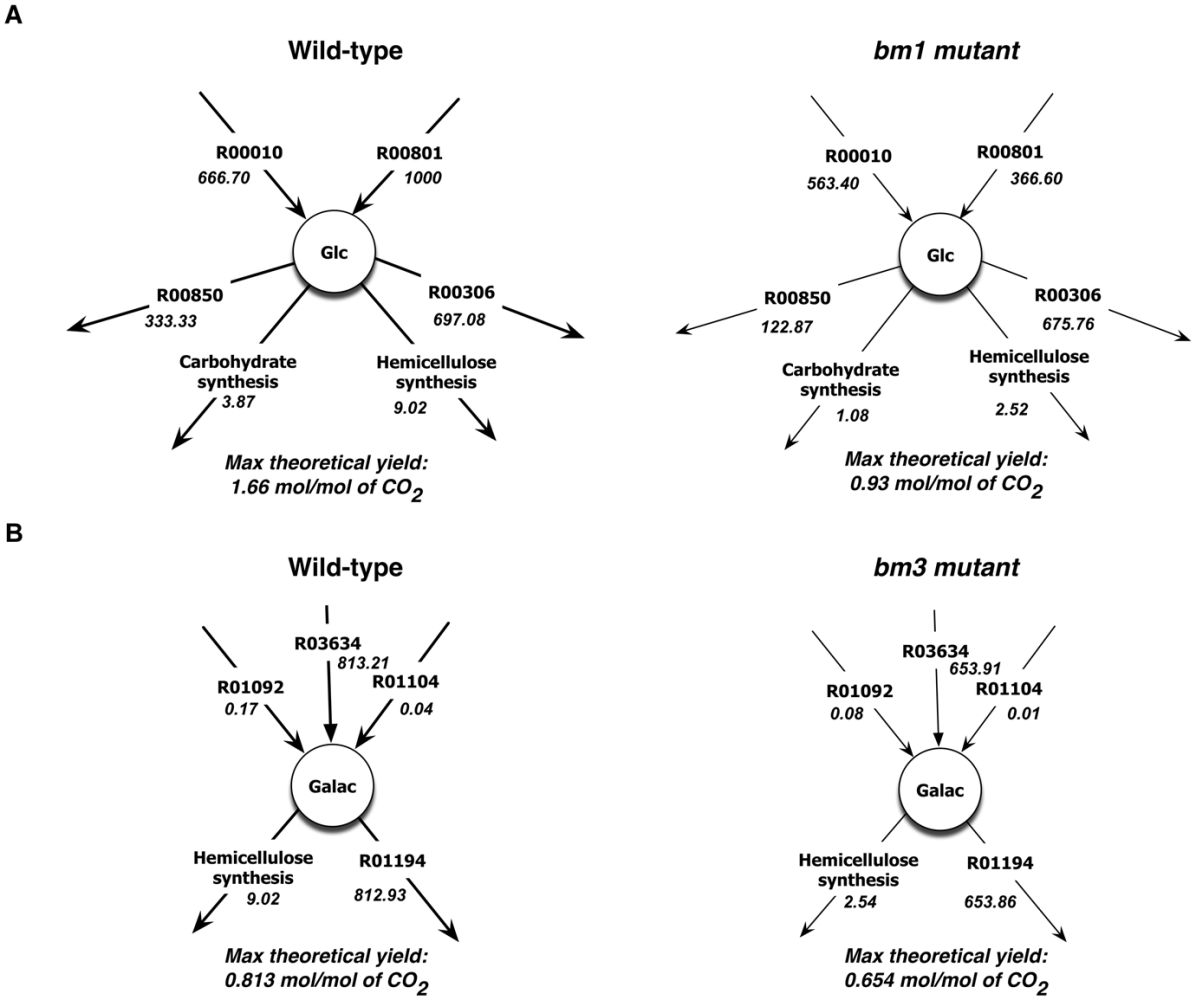
Maximum theoretical yields of (a) glucose and (b) galactose for wild-type vs *bm1* mutant and wild-type vs *bm3* mutant, respectively. Here the numeric values represent reaction fluxes and have the unit of mM/gDW-h.

In Figure 6 we highlight two cases that describe the availability of glucose and galactose to cell wall for mutants *bm1* and *bm3*, respectively. ‘Carbohydrate synthesis’ and ‘Hemicellulose synthesis’ are aggregate reactions that describe the utilization ratios of sugar molecules such as arabinose, fructose, galactose, glucose ribose, mannose, sucrose, and xylose for the production of carbohydrate and hemicellulose present in the plant cell wall. For simplicity, we have simulated the model under the photosynthetic condition where CO_2_ can be uptaken with a maximum allowable rate of 1000 mM/gDW-h along with photons in excess. In Figure 6a, wild-type and *bm1* mutant flux values for reactions involving glucose as reactant including ‘Carbohydrate synthesis’, ‘Hemicellulose synthesis’, ‘Alpha,alpha-trehalose glucohydrolase’ [R00010], ‘Sucrose glucohydrolase’ [R00801], ‘Sn-Glycerol-3-phosphate: D-glucose 6-phosphotransferase’ [R00850] and ‘Cellobiose glucohydrolase’ [R00306], are highlighted. For the wild-type case, the maximum theoretical yield of glucose is predicted to be 1.66 moles/mole of CO_2_ but it is reduced to 0.93 moles/moles of CO_2_ for the *bm1* mutant. The reduced capability of the *bm1* mutant to direct flux towards ‘Carbohydrate synthesis’ and ‘Hemicellulose synthesis’ implies that less glucose is available for the formation of cell wall components which is consistent with the experimental finding of Table 6.

Figure 6b contrasts the wild-type and *bm3* mutant maximum theoretical yields for all reactions involving galactose including ‘Hemicellulose synthesis’, ‘ATP: D-galactose 1-phosphotransferase’ [R01092] and ‘Galactosylglycerol galactohydrolase’ [R01104], ‘3-O-alpha-D-Galactosyl-1D-myo-inositol galactohydrolase’ [R01194] and ‘alpha-galactosidase’ [R03634]. A reduction of the maximum theoretical yield of galactose from 0.81 to 0.65 moles/mole of CO_2_ for the *bm3* mutant is observed. In addition, the maximum theoretical yield for reaction ‘Hemicellulose synthesis’ decreases by 4-fold compared to wild-type in line with the experimental finding. However, the experimentally observed increase of glucose availability in mutant *bm3* and xylose availability for both *bm1* and *bm3* mutants are in contrast with the model predictions (see Table 6). As reported by Guillaumie et al (2007) several gene expression levels were changed during *bm1* and *bm3* mutations implying that additional regulatory constraints may be needed to capture these changes.

## Discussion

Maize, apart from its central role a food crop, is also a promising plant biomass target for cellulosic biofuels production. Plant cell wall cellulose, hemicellulose and lignin polymers are major contributors of plant biomass [48], [58]. Therefore, controlling the amount and composition of cell wall polymers is important in developing cellulosic maize for biofuel production. In cell wall, lignin provides rigidity by forming a matrix where cellulose and hemicellulose are imbedded via cross-linking bonds [53], [59]. This makes digestion of cellulose and hemicellulose by microbial enzymes (i.e., cellulases) difficult during dilignification, one of the critical steps in cellulosic biofuel production [60]. Many genetic modification strategies have been explored to improve maize food crop and/or biofuel characteristics. For example, cellulosic biomass yield improvements have been pursued before by altering the lignin content and composition [61], [62], genetically manipulating the cellulose biosynthetic pathway [63] and over-expressing the gene encoding phosphoenolpyruvate carboxylase (PEPC) to improve CO_2_ fixation rate [64]. At the same time, grain yield enhancements have been attempted by up-regulating ADP-glucose pyrophosphorylase (AGP) that catalyzes the rate limiting step in starch synthesis [65].

Unfortunately, existing genetic engineering strategies to reduce lignin content are problematic as lignin reductions are usually achieved at the expense of plant viability and fitness [60]. It is becoming widely accepted that focusing on a single pathway at a time without quantitatively assessing the system-wide implications of the genetic disruptions may be responsible for not preserving the agronomic properties of the plant. By accounting for both primary and some secondary metabolism pathways of maize, *Zea mays i*RS1563 can be used to explore *in silico* the effect of genetic modifications aimed at plant cell wall modification and/or starch storage on the overall metabolic state of the plant (e.g., biomass precursor availability, cofactor balancing, redox state, etc.). Moving a step further, the use of computational strain optimization techniques [66], [67] can be customized for engineering plant metabolism. By taking full inventory of plant metabolism optimal gene modifications could be pursued for a variety of targets in coordination with experimental techniques. These may include (i) increase cellulose and hemicellulose production, (ii) starch yield, (iii) tolerance against biotic stress (e.g., fungal elicitation), or (iv) disruption of the production of lignin subunits (H/G/S) while enhancing the production of easily digestible lignin precursor (e.g., rosmarinic acid, conferyl ferulate, tyramine conjugates, etc).

In this paper, we introduced the first comprehensive genome-scale metabolic model (*Zea mays i*RS1563) for maize metabolism. The model meets (or exceeds) the quality and completeness criteria set out [68], [69] for genome-scale reconstructions. In analogy to the human genome-scale model Recon 1 [70], *Zea mays i*RS1563 can be viewed as a mathematically structured database enabling systematic studies of maize metabolism.185 of unique to maize reactions accounting for a fraction of secondary metabolism were delineated. As a by product of this effort a more up-to-date version of AraGEM [12] was constructed including GPR associations. Comparisons between *Zea mays i*RS1563 and maize C4GEM also revealed the detail in description of primary and secondary metabolism. Model predictions of *Zea mays i*RS1563 for two widely occurring maize Mendelian mutants were tested against experimental observations with very good agreement in the direction of changes. By making use of high throughput enzymatic assays, proteomic and transcriptomic data across different parts of the maize plant, *Zea mays i*RS1563 could serve as the starting point for the development of tissue-specific maize models [20], [71], [72]. Furthermore, *Zea mays i*RS1563 could also serve as the stepping stone for the development of genome-scale models for other important C_4_ plants such as Sorghum and switch grass.

## Materials and Methods

A number of recent publications [15], [27], [68] have outlined the general steps necessary for the metabolic reconstruction process. In the following section, we highlight the specific methods used in the reconstruction of *Zea mays i*RS1563 and subsequent model simulations in more detail.

### Model reconstruction

The maizesequence database [10] provided the filtered gene set (FGS) which has been generated from the working gene set upon removing pseudogenes and low confidence hypothetical models. The FGS of B73 maize genome (release 4a.53) was downloaded from maizesequence database on February 17, 2010. Once maize genes were obtained, we used sequence comparison tools [73] such as stand-alone BLAST (version 2.2.22, NIH) and BLAST+ (version 2.2.22, NIH) for performing homology comparisons. Marvin (version 5.3.3, ChemAxon Kft) was used to calculate the average micro-species charge to determine the net charge of individual metabolites at pH 7.2 assumed for all organelles. In the final step of the model reconstruction, we implemented GapFind and GapFill [37] for analyzing and subsequently restoring metabolic network connectivity.

### Model simulations

Flux balance analysis (FBA) [74] was employed both in model validation and model testing phases. *Zea mays i*RS1563 was evaluated in terms of biomass production under three standard physiological scenarios: photosynthesis, photorespiration, and respiration. Flux distributions for each one of these states were approximated using FBA:
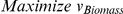
Subject to
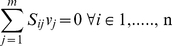
(1)

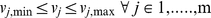
(2)Here, *S_ij_* is the stoichiometric coefficient of metabolite *i* in reaction *j* and *v_j_* is the flux value of reaction *j*. Parameters *v_j,min_* and *v_j,max_* denote the minimum and maximum allowable fluxes for reaction *j*, respectively. As mentioned in Table 4, the three physiological states were represented via modifying the relevant minimum or maximum allowable fluxes and the following constraints:
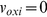
(3)

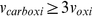
(4)

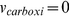
(5)where *v_Biomass_* is the flux of biomass reaction and *v_oxi_* and *v_carboxi_* are the fluxes of carboxylation and oxidation reactions associated with enzyme RUBISCO. For photosynthesis and photorespiration, constraints (3) and (4) were respectively included in the linear model, whereas for respiration both constraints (3) and (5) were included.

Once the model was validated, it was further tested for two maize mutants (i.e., *bm1* and *bm3*) under the photosynthetic condition. The following two constraints were included individually in the linear model to represent the mutants:
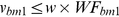
(6)

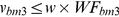
(7)Here, *w* represents the percent of residual activity of 10%. *v_bm1_* and *v_bm3_* are the fluxes of reactions catalyzed by CAD and COMT, respectively and *WF_bm1_* and *WF_bm3_* are the corresponding wild-type flux values under the photosynthetic condition.

CPLEX solver (version 12.1, IBM ILOG) was used in the GAMS (version 23.3.3, GAMS Development Corporation) environment for implementing GapFind and GapFill [37] and solving the aforementioned optimization models. All computations were carried out on Intel Xeon E5450 Quad-Core 3.0 GH and Intel Xeon E5472 Quad-Core 3.0 GH processors that are the part of the lionxj cluster (Intel Xeon E type processors and 96 GB memory) of High Performance Computing Group of The Pennsylvania State University.

## Supporting Information

File S1 *A. thaliana i*RS1597 model along with established GPR for AraGEM.
(XLS)

File S2 Sequential stages of *Zea mays i*RS1563 model development.
(XLS)

File S3 Results from BLASTp searches against NCBI nr database.
(XLS)

File S4 Biomass equation, added reactions for restoring biomass production under all three physiological conditions and unique reactions in *Zea mays i*RS1563 model.
(XLS)

File S5 *Zea mays i*RS1563 model.
(XLS)

File S6 SBML file of *Zea mays i*RS1563 model.
(XML)

File S7 Literature data used for testing *Zea mays i*RS1563 model.
(XLS)
